# Adherence to cervical cancer screening varies by human papillomavirus vaccination status in a high-risk population

**DOI:** 10.1016/j.pmedr.2015.07.011

**Published:** 2015-07-31

**Authors:** Christopher A. Paynter, Benjamin J. Van Treeck, Inge Verdenius, Agnes W.Y. Lau, Twinkle Dhawan, Kayla A. Lash, Elizabeth A. Bergamini, Chiazotam N. Ekekezie, Amna M. Hilal, Kristen N. James, Sadie Alongi, Sean M. Harper, Aaron J. Bonham, Kathy B. Baumgartner, Richard N. Baumgartner, Diane M. Harper

**Affiliations:** a University of Missouri—Kansas City School of Medicine, Kansas City, MO, USA; b Mayo School of Graduate Medical Education, Rochester, NY, USA; c Radboud University, Nijmegen, The Netherlands; d Rutgers-New Jersey Medical School, Newark, NJ, USA; e Jackson Memorial Hospital, Miami, FL, USA; f University of Texas Medical School Houston, TX, USA; g St. Louis University, St. Louis, MO, USA; h Brown University, Providence, RI, USA; i North Shore-LIJ Health System, New York, NY, USA; j University of Louisville, Department of Mathematics, Louisville, KY, USA; k McMaster University, Hamilton, ON, Canada; l University of Louisville, School of Public Health and Information Sciences, Department of Epidemiology and Population Health, Louisville, KY, USA; m University of Louisville, School of Medicine, Department of Family and Geriatric Medicine, Louisville, KY, USA; n University of Louisville, School of Medicine, Department of Obstetrics and Gynecology, Louisville, KY, USA; o University of Louisville, School of Public Health and Information Sciences, Department of Health Promotion and Behavioral Health Sciences, Louisville, KY, USA; p University of Louisville, Speed School of Engineering, Department of Bioengineering, Louisville, KY, USA

**Keywords:** HPV4, means quadrivalent HPV vaccine Gardasil™, HPV4, Compliance, Cervical cancer screening, Cervical cancer, Women

## Abstract

Cervical cancer screening has reduced the incidence of cervical cancer over the past 75 years. The primary aim of this study was to determine if women receiving Gardasil™ (HPV4 vaccine) participated in future cervical cancer screening at the same rate as that observed for unvaccinated women matched on birth year and health care campus. This is a retrospective cohort study of subjects selected from 27,786 females born from 1980 to 1992 who received health care in the Truman Medical Center safety net health system in Kansas City Missouri, USA. 1154 women 14–26 years old who received at least one dose of HPV4 vaccine between 2006 and 2009 were chosen at random from the vaccine records. 1154 randomly chosen unvaccinated women were age and health campus matched to the vaccinated women and all were followed until July 1, 2013. Women who were screened after 21 years and received three vaccine doses before 21 years, had the lowest screening rate of 24%. Their only predictive factor for screening, compared to the unvaccinated, was being closer to 21 years than 14 years at vaccination (aOR = 1.71 95% CI: 1.45, 2.00). Women vaccinated with three doses and screened at or after 21 years had the highest screening rate of 84% predicting a six-fold increase in screening participation over no vaccine received (aOR = 5.94 95% CI: 3.77, 9.35). Our results suggest that women who receive HPV4 vaccination closer to 21 years, not 14, are more likely to participate in cervical cancer screening in an underserved US population.

## Highlights

• Screening rates are extremely low (24%) among those targeted for HPV vaccination.
• Screening rates are 84% if vaccinated at 21 years or older.
• Only HPV vaccination at ages closer to 21 years predicts screening participation.
• 3 dose recipients are more likely than unvaccinated to continue screening after NILM.
• Only 50% of vaccinated women continue to screen after a NILM result.

## Introduction

Much work has been published on increasing the uptake and completion rates of HPV vaccination with the hopes that cervical cancer may be prevented in the future (Cullen et al., 2014). While these efforts are commendable, screening programs are the only proven method of reducing cervical cancer (de Blasio et al., 2012, Harper et al., 2010, Kulasingam et al., 2007). The longstanding effectiveness of cytology screening derives from high participation rates (Centers for Disease Control and Prevention, 2012, Committee on Practice Bulletins—Gynecology, 2012), and most recently its effectiveness increases by the change to include HPV testing (Moyer et al., 2012, Huh et al., 2015).

Women at the highest risk for cervical cancer are those who are underserved and uninsured, and who seek care in the safety net health care system (Lewin, 2000, Freeman and Wingrove, 2005). The National Cancer Institute (NCI) Center to Reduce Cancer Health Disparities (CRCHD) postulates that cervical cancer is an indicator of larger health system concerns, a bellwether for other health care vulnerabilities (Freeman and Wingrove, 2005, Scarinci et al., 2010). By understanding women's behavior in high-risk populations after HPV vaccination towards future cervical cancer screening participation, we may be able to reduce cervical cancer and its associated vulnerabilities (Freeman and Wingrove, 2005).

A high intent to participate is noted for those adults 21 years and older both vaccinated and unvaccinated (Alexander et al., 2014, Anhang Price et al., 2011). On the other hand, adolescents noted a much lower intent to screen if no vaccine or fewer than three doses were received (Bowyer et al., 2014). The primary aim of this study was to determine if HPV4 vaccination was a predictor of adherence to cervical cancer screening in an underserved population. The secondary aim is to explore whether HPV4 vaccination predicts whether women with NILM screen results continue to participate in routine screening.

## Materials and methods

In this retrospective cohort study, subjects were selected from the 27,786 young women aged 14–26 years of age seen in the Truman Medical Center (TMC) safety net health care system in Kansas City, MO, USA between July 1, 2006 and October 1, 2009, and followed through July 1, 2013, when the youngest vaccinated would be at 21 years of age, the US recommended screening age. TMC provides health care at two campuses for the underinsured, uninsured or low income patients who are at high risk for adverse health outcomes, including cervical cancer. Study subjects were randomly chosen among those who received at least one dose of the quadrivalent HPV vaccine (HPV4) between July 1, 2006 and October 1, 2009 in the TMC HPV Vaccination Program and had at least one health care visit at TMC after vaccination and who would be of screening age as of July 1, 2013. Vaccine ascertainment was based on patient logs maintained for vaccine accountability, billing records, and the Electronic Medical Record (EMR).

Women who had not been vaccinated during the baseline window or follow-up period were matched to those vaccinated by year of birth and health care system campus at which they received health care. Likewise, they had at least one health care visit after the first vaccination date of their match and were of screening age by July 1, 2013. Age at first vaccine dose in this group of unvaccinated women refers to the age at which the matched vaccinated woman received her first HPV4 dose. Age matching was necessary to create the comparator non-vaccinated cohort because cervical cancer screening is age dependent.

EMR data included self-identified race/ethnicity, pregnancy history, health campus, and related screening data. TMC had no organized call/recall program to notify patients of a need for cervical cancer screening, something common in the US. The cytology screening nomenclature adhered to the 2001 Bethesda system (Solomon et al., 2002) which included negative for intraepithelial lesion or malignancy (NILM). The observed cervical cancer screening guidelines were those in effect in 2009 (American Congress of Obstetricians and Gynecologists Committee Meeting Number 431, 2009), where the recommended age of screening initiation was 21 years and, if NILM, screening was recommended at 3-year intervals. Screening is defined as too early if occurring before 21 years. This study was approved by the UMKC Adult Health Sciences Institutional Review Board (#12-351) and all required protections concerning the de-identification of data were observed with all data de-identified prior to analysis.

## Statistical analysis

The age threshold used in this analysis was 21 years to correspond to the age of initiation of cervical cancer screening (screening eligibility); hence, both age at first vaccination and age of first screening thereafter were dichotomized to younger than 21 years vs. 21 years or older.

The study was designed to have 80% power to detect an absolute difference of 6% between screening rates among those receiving at least one dose of HPV4 vs those with no vaccination using a Pearson's chi-square test with a two-sided significance level of 0.05. We calculated that the analysis would require 1090 in each group of vaccinated and unvaccinated women, which we increased to 1154 in each group to allow for missing data on confounding factors abstracted from the EMR. For all continuous variables, the mean and standard error in each group are reported. We derived the Cochran–Armitage test for trend by using a transformation of the linear-by-linear association provided by SPSS (IBM Corp. (Released, 2011)).

We used conditional binary logistic regression to predict participation in cervical cancer screening by number of HPV4 vaccine doses received (0–3) as well as demographic and obstetrical descriptors using the unadjusted and adjusted odds ratios (ORs and aORs, respectively) and 95% confidence intervals (CIs). These analyses were performed with Statistica version 12 (StatSoft, 2013). A two-tailed p-value of less than 0.05 was considered statistically significant for all analyses, except the univariate regression, where p < 0.10 was used to exclude nonsignificant parameters.

## Results

Of the 2308 women followed, 38% (875/2308) were too young for screening participation (< 21 years) at the time of first or matched vaccination (non-screen eligible); 62% (1433/2308) were ≥ 21 years. The population descriptors in Table 1 show that the non-screen eligible vaccinees were significantly younger than the screen eligible vaccinees at age of first screening (20.6 yrs (SE 0.09) vs. 25.1 years (SE 0.07), p < 0.001). There was no difference between the non-screen eligible and screen eligible in distribution of doses of vaccine. Descriptors by vaccination status are in the Supplementary Table.

Participation in cervical cancer screening varied widely by age of first or matched vaccine dose. Overall, 55% (1276/2308) of the total population studied participated in cervical cancer screening (Table 2) with women who received any number of vaccine doses participating in screening significantly more often than those without vaccination (59% (682/1154) vs. 51% (594/1154), p < 0.001).

Among the women who received the first or matched vaccine dose at less than 21 years, a significant proportion screened too early (< 21 years) than on time (≥ 21 years): 29% (258/875) vs 25% (217/875), p < 0.05. Prenatal screening among those less than 21 years (29% (75/258)) occurred significantly more often among unvaccinated women compared to vaccinated women (47% (54/114) vs. 15% (21/144), p < 0.001).

Among women whose first or matched vaccine dose was ≥ 21 years, vaccinated women screened significantly more often than unvaccinated women (62% (428/694) vs. 50% (373/733), p < 0.001). In addition, the more vaccine doses a woman received, the higher the screening rate was (84% (175/209) vs. 62% (107/172) vs. 47% (146/313), p_trend: three-two-one dose_ < 0.001)).

Screening rates among women screened ≥ 21 years were significantly lower among women vaccinated before 21 years who received three doses compared to three dose recipients vaccinated ≥ 21 years (24% (40/161) vs. 84% (175/209), p < 0.001).

## Predictors of Cervical Cancer Screening

The binary logistic regression model predicted participation in cervical cancer screening using vaccination status, number of doses received, race and pregnancy history in univariate models. A multivariate model controlled for the significant univariate predictors of screening to indicate the most influential predictors (Table 3).

The model was stratified into three groups. The first modeling considered all women regardless of age of first vaccine dose or age of screening. Univariate predictors showed that women who had received at least one HPV4 dose were significantly more likely than unvaccinated women to participate in screening (OR = 1.36, 95% CI: 1.16, 1.61). More specifically, women who received two or three HPV4 doses were significantly more likely to participate in screening compared to women receiving no vaccine (OR = 1.46 (95% CI: 1.12, 1.83) and OR = 2.81 (95% CI: 1.04, 1.49), respectively); whereas, women receiving a single HPV4 dose had the same likelihood of screening participation as the unvaccinated women. Black women were 24% more likely to participate in screening than white women (OR = 1.24, 95% CI: 1.04, 1.49).

In the multivariate model, when controlling for vaccination status, number of doses received and race, receiving any number of doses of HPV4 was not a significant predictor of screening (aOR = 0.82 95% CI: 0.67, 1.02). Black women compared to white women (aOR = 1.29 95% CI: 1.07, 1.54) and only those receiving two or three doses compared to the unvaccinated (aOR = 1.80 95% CI: 1.34, 2.41, and aOR = 3.46 95% CI: 2.58, 4.65, respectively) were significantly more likely to participate in screening.

The second modeling considered those vaccinated younger than 21 years and screened ≥ 21 years, as would be an appropriate population cancer prevention plan. The closer to 21 years compared to 14 years the woman was at vaccination, the more likely she was to participate in screening (OR = 1.65 95% CI: 1.42, 1.92). Vaccinated compared to unvaccinated women were significantly more likely to participate in screening (OR = 1.55 95% CI: 1.11, 2.16), and women receiving two or three HPV4 doses were significantly more likely than the unvaccinated woman to participate in screening (OR = 2.08 95% CI: 1.30, 3.33, OR = 1.63 95% CI: 1.05, 2.53, respectively). In addition, Black women were 54% more likely to participate in screening than white women (OR = 1.54, 95% CI: 1.07, 2.22). In the multivariate model, when controlling for age at first vaccination, vaccine status, number of vaccine doses and parity, only age at first vaccination was significantly more likely to predict screening participation. Specifically, we see again that the closer to 21 years at first vaccination compared to 14 years, the more likely the woman participated in screening (aOR = 1.71, 95% CI: 1.45, 2.00).

The third modeling considered women vaccinated and screened ≥ 21 years. In this group, the age at first vaccine dose was not a significant predictor of participation in screening. Women with at least one vaccine dose were significantly more likely to participate in screening than unvaccinated women (OR = 1.58, 95% CI: 1.28, 1.95). Again, only those receiving two or three doses compared to no doses were significantly more likely to participate in screening (OR = 1.61 95% CI: 1.15, 2.27 and OR = 5.05 95% CI: 3.40, 7.49, respectively). In the multivariate model, when adjusting for vaccine status, number of doses, gravidity and parity, vaccinated women were significantly less likely to participate in screening than unvaccinated (aOR = 0.48, 95% CI: 0.36, 0.65), a flip because of the disproportionate weight of single dose recipients not participating in screening. Similarly, only those receiving two and three doses were significantly more likely to participate in screening than those receiving no doses (aOR = 2.02 95% CI: 1.36, 3.01 and aOR = 5.94 95% CI: 3.77, 9.35, respectively).

## Continued routine screening

The relationship between HPV4 vaccination and adherence with routine screening after receiving one NILM result is low. Overall, regardless of vaccine status, only 45% (442/977) of women with NILM screening results participated in a second routine screen (Table 4). There was significantly greater adherence to participating in a second screening among those who received three HPV4 doses compared to the unvaccinated woman (62% (127/205) vs. 40% (193/477), p < 0.001). Moreover, only 52% (105/201) of early NILM result screeners (< 21 years) returned for a second screen during the 6.5 year study follow-up, and only half were older than 21 years at this second routine screen. Nonetheless, screening participation among women receiving three doses was significantly decreased at the second round compared to the rate of initial screening (62% (127/205) vs 75% (277/470), p < 0.01).

Stratifying by age of first HPV4 vaccination, those younger than 21 years at first HPV4 dose had significantly lower routine second screen rates than those women whose first vaccine dose was at or older than 21 years (13% (11/78) vs 64% (81/147), p < 0.05).

Predictors of continued screening after an initial NILM result are presented in Table 5. Among all women, while vaccine status predicted significantly lower chances of routine screening than unvaccinated women after an initial NILM result (OR = 0.68 (95% CI: 0.53, 0.88)), women receiving three doses of HPV4 were significantly more likely to adhere to routine screening than the unvaccinated woman (OR = 2.40 (95% CI: 1.71, 3.35)). The multivariate logistic regression resulted in older age and receiving three doses of HPV4 being the only predictors for adherence to follow up routine screening (aOR = 1.08 (95% CI: 1.03, 1.13) and 2.34 (1.51, 3.61) respectively).

Among the young vaccinees screening ≥ 21 years, there were two strong predictors for routine screening adherence. The closer the age of vaccination was to 21 years from 14 years, the greater the likelihood of adherence to future screening (OR = 1.83 (95% CI: 1.16, 2.88)). Similarly, receiving three doses compared to none was a significant predictor of adherence to routine screening (OR = 2.72 (1.06, 6.99)). In multivariate modeling, both age of vaccination and receiving three HPV4 doses retained their significance, indicating those who received vaccine closer to 21 years and who received all three doses compared to none, were significantly more likely to adhere to continued routine screening (aOR = 2.06 (95% CI: 1.28, 3.32) and aOR = 3.46 (95% CI: 1.25, 9.59), respectively).

Finally, among women vaccinated and screened ≥ 21 years, women were significantly more likely to adhere to routine screening if they had at least one vaccine dose compared to none (OR = 1.46 (95% CI: 1.06, 2.01)), but specifically had received all three doses of vaccine (OR = 2.25 (95% CI: 1.47, 3.44)) compared to none. In the multivariate model, when controlling for these variables, women who received three HPV4 doses were significantly more likely than the unvaccinated women to adhere to routine screening (aOR = 1.84 (1.17, 2.89)).

## Discussion

We report that participation in cervical cancer screening among the women choosing no HPV vaccination in this underserved, high-risk population is significantly lower (50%) than would be anticipated from US Behavioral Risk Factor Surveillance System (BRFSS) responses, which in 2012 were 86% for women 21–29 years old (Benard et al., 2007), and 81% from the US National Health Interview Survey (NHIS) study (Bowyer et al., 2014). An Australian report of women choosing no HPV vaccination during an active HPV vaccine program also showed that unvaccinated adult women screened at similarly low rates: 20–24 year olds at 48% and 25–29 year olds at 59%, despite an organized notification system for screening (Budd et al., 2014). While our population historically has had the poorest screening rates in the US, with the advent of HPV vaccines, we have not seen a decrease in the baseline rate, whereas both the UK and the Scottish NHS reported much lower screening rates among their unvaccinated women at 39% and 30% during active HPV vaccination programs compared to prior to the vaccine programs (Beer et al., 2014 Apr 1, Pollock et al., 2014, July 18, 2015).These low screening rates are concerning not only because these women are the ones choosing no HPV vaccination, but because most cervical cancer occurs in those with no screening or screening at prolonged intervals which is significantly more common among high-risk women (Drolet et al., 2013). Including the unknown duration of HPV vaccine efficacy, and that none of the HPV vaccines prevents all cervical cancers, the need for promoting screening participation during HPV vaccination programs is essential.

Vaccinated women in our high risk population participated in screening more often than the unvaccinated, and in a manner dependent on the number of HPV4 vaccine doses received. Women receiving three doses ≥ 21 years had the best screening rate of 84%, equivalent to the Healthy People 2020 baseline screening rate (Healthy People, 2020). It is plausible that the three visits for vaccination ≥ 21 years provide an increased opportunity to discuss and participate in screening, as we saw significantly lower screening rates among those receiving only one (47%) or two (62%) doses. In addition, vaccinated women were more often screened than unvaccinated women in a Danish and two US survey studies (Sauer et al., 2015, Beer et al., 2014 Apr 1, Baldur-Felskov et al., 2014 Mar), whereas the Australian study showed lower screening rates among the vaccinated (Budd et al., 2014).

Nevertheless, the population targeted for vaccination and screening is that whose vaccinations are separated by many years from screening. In our study, participation rates in screening among the women vaccinated younger than 21 years and screened ≥ 21 years dropped to an unacceptably low rate of 24%, not different from the unacceptably low 26% screening rate of unvaccinated women in this same cohort. It is important to call out that the only predictor of screening participation in this targeted age group of women was an age closer to 21 than 14 years. This is particularly important to note as we have shown that three dose HPV vaccination at screen eligible ages (≥ 21 years) predicts nearly a six-fold increase in screening compared to the unvaccinated in our high-risk population.

We also document inappropriate screening in that 54% (258/475) of screened women, vaccinated younger than 21 years, were screened inappropriately before 21 years. While some would posit that any screening after vaccination is a successful outcome, we believe that for those young adolescents to whom HPV vaccination is targeted, a 29% (258/875) screen rate before reaching 21 years wastes resources for no measurable health outcome. Participating in screening too early is harmful: cytology is not sensitive for CIN 3 detection at young ages, the examination and screening is costly, and the speculum examination creates unproductive anxiety in the young woman (Committee on Practice Bulletins—Gynecology, 2012, Reddy and Wasserman, 1997, Snodgrass and Naugler, 2014).

Moreover, a NILM result may inappropriately reassure a young woman that she does not have cancer and dissuade her from future screening (Henderson et al., 2011). Failure-to-screen behavior is a predictor of cervical cancer; Leyden et al (Leyden et al., 2005) showed that 31% of the cervical cancers diagnosed in women 16–39 years old were associated with no screen in the prior 3 years. Our data show that there is a high risk of failure to continue screening in the presence of a NILM result after early vaccination. In fact, the only predictors of participation in routine screening after an initial NILM result for the targeted young vaccinee who waited until 21 years or older for screening was an age of vaccine closer to 21 years than 14 years, and receiving three vaccine doses compared to no doses.

The limitations of our study include the fact that it is a retrospective cohort study where screening call/recall is not used in an organized program. All age-appropriate women presenting to our safety net health care system were offered HPV vaccination during the time frame of the study, eliminating unequal access to the vaccine as a source of bias. Likewise, all women reached the screening age of 21 years by study end and had had at least one other health care visit for health care concerns by study end, minimizing the possibility that the women chose to be screened outside of this health care system, which is a possibility, although unlikely.

Information biases may be present, as well, in both screening and vaccination ascertainment. The EMR may include unknown recording errors that may have produced misclassification of screening status. In addition, while the vaccination logs for all administration of HPV4 vaccines may exclude doses given but not recorded, the pharmacy log of doses given completely agrees with the vaccination log making it highly unlikely that vaccine doses were misclassified in this catchment population. Our study is observational and therefore identifies only statistical associations which may or may not reflect causal relationships. A strength of our study, however, is the relatively large sample size in a captured safety net population.

## Conclusions

In summary, we strongly recommend continuing active HPV vaccination programs among women ≥ 21 years at the same time as screening. Because our results show that adolescent HPV vaccination efforts have not resulted in adequate screening participation, we recommend vigorous counseling for screening at the time of vaccination.

The following are the supplementary data related to this article.

Supplementary Table Vaccinated vs. non-vaccinated age and health care campus matched female descriptors.

## Conflict of interest statement

None of the authors have a conflict of interest to report.

## Figures and Tables

**Table 1:** Characteristics of the study population.

	Non-screen eligible	Screen eligible
	Age at first or matched vaccine dose < 21 yrs	Age at first or matched vaccine dose ≥ 21 yrs
	N = 875	N = 1433
Age at first screening after first vaccine dose, yrs, mean (SE), range	**20.6 (0.09)** 14–27 yrs	**25.1 (0.07)** 21–35 yrs
*Race/ethnicity, n (%)*		
White	314 (36)	500 (35)
Black	470 (54)	759 (53)
Hispanic	57 (6)	95 (6)
Other	34 (4)	78 (6)

*Gravidity, n (%)*		
n = 0	239 (32)	196 (17)
n ≥ 1	**505 (68)**	**972 (83)**

*Parity, n (%)*		
n = 0	272 (37)	258 (22)
n ≥ 1	**472 (63)**	**908 (78)**

*Total number of doses received, n (%)*		
n = 0	416 (48)	738 (51)
n = 1	181 (21)	313 (22)
n = 2	118 (13)	172 (12)
n = 3	161 (18)	209 (15)
Three on-time⁎ doses completed, n (%)	110 (68)	150 (72)

SE means standard error.

Bold indicates statistical significance at p < 0.001.

⁎ On-time dosing of HPV4 was defined as dose 2 is ≥ 4 weeks and ≤ 26 weeks from dose 1; dose 3 is > 24 weeks and ≤ 52 weeks from dose 1 and dose 3 is ≥ 12 weeks from dose 2.

**Table 2:** Participation in Cervical Cancer Screening within 7 years of the first HPV4 dose.

	First or Matched Vaccine Dose < 21 years N = 875				First or Matched Vaccine Dose ≥ 21 years N = 1433		Total Population N = 2308	
	Screened < 21 years	Screened ≥ 21 years	All Screened	Not Screened	Screened ≥ 21 years	Not Screened	Screened	Not Screened
	N = 258	N = 217	N = 475	N = 400	N = 801	N = 632	N = 1276	N = 1032
**No Vaccine, n/N (%)**	114/415 (27)	107/415 (26)	221/415 (53)	194/415 (47)	373/739 (50)	366/739 (50)	*594/1154 (51)*	560/1154 (49)
**Any Vaccine Doses, n/N (%)**	144/460 (31)	110/460 (24)	254/460 (55)	206/460 (45)	428/694 (62)	266/694 (38)	*682/1154 (59)*	472/1154 (41)
Three Doses	62/161 (39)	**40/161 (24)**	102/161 (63)	59/161 (37)	**175/209 (84)**	34/209 (16)	277/370 (75)	93/370 (25)
Two Doses	34/118 (29)	35/118 (30)	69/118 (58)	49/118 (42)	107/172 (62)	65/172 (38)	176/290 (61)	114/290 (39)
One Dose	48/181 (27)	35/181 (19)	83/181 (46)	98/181 (54)	146/313 (47)	167/313 (53)	229/494 (46)	265/494 (54)

Bold indicates that among those receiving three doses and participating in screening at 21 years or older, the screening rate among those women who received their first or matched vaccine dose at non-screen eligible ages was significantly lower than the screening rate among women who received their first or matched vaccine dose at a screen-eligible age, p < 0.001. Italicized indicates significantly more women participated in screening if they were vaccinated than unvaccinated, p < 0.001.

**Table 3:** Predictors of Cervical Cancer Screening Participation by Screen Eligible Age at First Vaccine Dose.

	All ages regardless of age of vaccination or screening N = 2308		< 21 years at first vaccine and screened at ≥ 21 years N = 617		≥ 21 years for vaccination and screening N = 1433	
	OR (95% CI)	aOR (95% CI)	OR (95% CI)	aOR (95% CI)	OR (95% CI)	aOR (95% CI)
Age at first vaccination	1.02 (0.99, 1.05)		**1.65 (1.42, 1.92)**	**1.71 (1.45, 2.00)**	0.99 (0.94, 1.06)	
Vaccination Status						
No vaccination	Referent	Referent	Referent	Referent	Referent	Referent
At least one HPV4 dose	**1.36 (1.16, 1.61)**	0.82 (0.67, 1.02)	**1.55 (1.11, 2.16)**	1.00 (0.62, 1.63)	**1.58 (1.28, 1.95)**	**0.48 (0.36, 0.65)**
Number of Doses Received						
n = 0	Referent	Referent	Referent	Referent	Referent	Referent
n = 1	0.81 (0.66, 1.00)	1.0	1.18 (0.75, 1.85)	1.0	0.86 (0.66, 1.12)	1.0
n = 2	**1.46 (1.12, 1.83)**	**1.80 (1.34, 2.41)**	**2.08 (1.30, 3.33)**	1.75 (0.99, 3.08)	**1.61 (1.15, 2.27)**	**2.02 (1.36, 3.01)**
n = 3	**2.81 (2.16, 3.65)**	**3.46 (2.58, 4.65)**	**1.63 (1.05, 2.53)**	1.50 (0.84, 2.60)	**5.05 (3.40, 7.49)**	**5.94 (3.77, 9.35)**
Race						
White	Referent	Referent	Referent	Referent	Referent	
Black	**1.24 (1.04, 1.49)**	**1.29 (1.07, 1.54)**	**1.54 (1.07, 2.22)**	1.36 (0.89, 2.08)	1.18 (0.94, 1.49)	
Hispanic	0.98 (0.69, 1.39)	1.08 (0.76, 1.53)	1.20 (0.59, 2.48)	1.05 (0.49, 2.28)	0.73 (0.47, 1.13)	
Other	0.97 (0.66, 1.44)	0.97 (0.65, 1.46)	1.31 (0.54, 3.16)	1.30 (0.46, 3.62)	0.99 (0.61, 1.59)	
Gravidity						
n = 0	Referent		Referent		Referent	Referent
n ≥ 1	1.04 (0.83, 1.30)		1.46 (0.99,2.14)		**0.57 (0.40, 0.80)**	0.88 (0.46, 1.69)
Parity						
n = 0	Referent		Referent	Referent	Referent	Referent
n ≥ 1	0.96 (0.78, 1.18)		**1.51 (1.05, 2.81)**	0.86 (0.56, 1.33)	**0.59 (0.44, 0.80)**	0.76(0.42, 1.34)

OR means odds ratio; aOR means adjusted OR for significant univariate predictors. Bold font indicates statistical significance. Odds ratios were adjusted for significant univariate parameters within each age group. 95% CI means 95% confidence interval.

**Table 4:** Subsequent Routine Cervical Cancer Screening among Women with an initial Negative for Intraepithelial Lesions or Malignancy (NILM) Result.

	Second Routine Screen							
	First or Matched Vaccine Dose < 21 years N = 365				First or Matched Vaccine Dose ≥ 21 years N = 612		Total Population N = 977	
	1^st^ and 2^nd^ Screen < 21 yrs	1^st^ and 2^nd^ Screen ≥ 21 yrs	All 2^nd^ Screens	No 2^nd^ Screen	2^nd^ Screen	No 2^nd^ Screen	2^nd^ Screen	No 2^nd^ Screen
	N = 48	N = 39	N = 144	N = 221	N = 298	N = 314	N = 442	N = 535
**No Vaccine, n/N (%)**	18/172 (10)	17/172 (10)	59/172 (34)	113/172 (66)	134/305 (44)	171/305 (56)	*193/477 (40)*	284/477 (60)
**Any Vaccine doses, n/N (%)**	30/193 (16)	22/193 (11)	85/193 (44)	108/193 (56)	156/299 (52)	143/299 (48)	249/500 (50)	251/500 (50)
Three Doses	12/78 (15)	**11/78 (13)**	46/78 (59)	32/78 (41)	**81/127 (64)**	46/127 (36)	*127/205 (62)*	78/205 (38)
Two Doses	10/54 (19)	5/54 (9)	19/54 (35)	35/54 (65)	40/79 (51)	39/79 (49)	59/133 (44)	74/133 (56)
One Dose	8/61 (13)	6/61 (10)	20/61 (33)	41/61 (67)	43/101 (43)	58/101 (57)	63/162 (39)	99/162 (61)

Bold indicates statistically significant differences in appropriate aged screening for three doses of vaccine received, p < 0.001. Italicized indicates that women receiving three doses and whose first screening was NILM were significantly more likely to participate in a second screen than the unvaccinated, p < 0.001.

**Table 5:** Predictors of Second Cervical Cancer Screening Participation after first NILM result.

	All ages regardless of age of vaccination or first screening N = 977		< 21 years at first vaccine and first screened at ≥ 21 years N = 164		≥ 21 years for vaccination and screening N = 612	
	OR (95% CI)	aOR (95% CI)	OR (95% CI)	aOR (95% CI)	OR (95% CI)	aOR (95% CI)
Age at first vaccination	**1.05 (1.00, 1.10)**	**1.08 (1.03, 1.13)**	**1.83 (1.16, 2.88)**	**2.06 (1.28, 3.32)**	1.01 (0.92, 1.11)	
Vaccination Status						
No vaccination	Referent	Referent	Referent		Referent	Referent
At least one HPV4 dose	**0.68 (0.53, 0.88)**	0.91 (0.62, 1.32)	1.31 (0.64, 2.71)		**1.46 (1.06, 2.01)**	1.0
Number of Doses Received						
n = 0	Referent	Referent	Referent	Referent	Referent	Referent
n = 1	0.94 (0.65, 1.35)	1.0	0.93 (0.33, 2.63)	0.80 (0.27, 2.32)	0.95 (0.60, 1.49)	0.88 (0.55, 1.41)
n = 2	1.17 (0.80, 1.73)	1.21 (0.75, 1.95)	0.81 (0.27, 2.43)	0.67 (0.22, 2.10)	1.31 (0.80, 2.15)	1.15 (0.68, 1.94)
n = 3	**2.40 (1.71, 3.35)**	**2.34 (1.51, 3.61)**	**2.72 (1.06, 6.99)**	**3.46 (1.25, 9.59)**	**2.25 (1.47, 3.44)**	**1.84 (1.17, 2.89)**
Race						
White	Referent		Referent		Referent	
Black	1.13 (0.86, 1.49)		1.15 (0.51, 2.58)		1.22 (0.87, 1.73)	
Hispanic	0.61 (0.35, 1.07)		0.33 (0.04, 2.85)		0.90 (0.38, 1.65)	
Other	0.53 (0.27, 1.04)		0.65 (0.07, 6.21)		0.64 (0.30, 1.38)	
Gravidity						
n = 0	Referent	Referent	Referent		Referent	Referent
n ≥ 1	**0.61 (0.45, 0.83)**	0.78 (0.40, 1.50)	1.13 (0.47, 2.74)		**0.52 (0.34, 0.80)**	**0.41 (0.18, 0.96)**
Parity						
n = 0	Referent	Referent	Referent		Referent	Referent
n ≥ 1	**0.63 (0.47, 0.85)**	0.78 (0.43, 1.44)	0.90 (0.39, 2.12)		**0.66 (0.45, 0.97)**	1.42 (0.67, 3.02)

OR means odds ratio; aOR means adjusted OR for significant univariate predictors. Bold indicates statistical significance. Odds ratios were adjusted for significant univariate parameters within each age group. 95% CI means 95% confidence interval

## References

[bb0065] Alexander N.M., Harper D.M., Comes J.C., Smith M.S., Heutinck M.A. (2014). Intent to participate in future cervical cancer screenings is lower when satisfaction with the decision to be vaccinated is neutral. PLoS One.

[bb0085] American Congress of Obstetricians and Gynecologists Committee Meeting Number 431 (2009). Routine pelvic examination and cervical cytology screening. Obstet. Gynecol..

[bb0070] Anhang Price R., Koshiol J., Kobrin S., Tiro J.A. (2011). Knowledge and intention to participate in cervical cancer screening after the human papillomavirus vaccine. Vaccine.

[bb0155] Baldur-Felskov B., Dehlendorff C., Munk C., Kjaer S.K. (2014 Mar). Early impact of human papillomavirus vaccination on cervical neoplasia—nationwide follow-up of young Danish women. J. Natl. Cancer Inst..

[bb0120] Beer H., Hibbitts S., Brophy S., Rahman M.A., Waller J., Paranjothy S. (2014 Apr 1). Does the HPV vaccination programme have implications for cervical screening programmes in the UK?. Vaccine.

[bb0100] Benard VB, Thomas CC, King J,Massetti GM,Doria-Rose VP,Saraiya M; Centers for Disease Control and Prevention (CDC). Vital signs: cervical cancer incidence, mortality, and screening - United States, 2007-2012. MMWR Morb Mortal Wkly Rep. 2014 Nov 7;63(44):1004-9. Available at: http://www.cdc.gov/mmwr/preview/mmwrhtml/mm63e1105a1. htm?s_cid = mm63e1105a1_w. Accessed April 1, 2015. PMC5779486 25375072

[bb0075] Bowyer H.L., Dodd R.H., Marlow L.A., Waller J. (2014). Association between human papillomavirus vaccine status and other cervical cancer risk factors. Vaccine.

[bb0115] Budd A.C., Brotherton J.M., Gertig D.M., Chau T., Drennan K.T., Chappeli G., Saville M. (2014). Cervical screening rates for women vaccinated against human papillomavirus. Med. J. Aust..

[bb0025] Centers for Disease Control and Prevention (2012). Cancer screening. MMWR.

[bb0030] Committee on Practice Bulletins—Gynecology (2012). ACOG practice bulletin number 131: screening for cervical cancer. Obstet. Gynaecol..

[bb0005] Cullen K.A., Stokley S., Markowitz L.E. (2014). Uptake of human papillomavirus vaccine among adolescent males and females: immunization information system sentinel sites, 2009–2012. Acad. Pediatr..

[bb0010] de Blasio B.F., Neilson A.R., Klemp M., Skjeldestad F.E. (2012). Modeling the impact of screening policy and screening compliance on incidence and mortality of cervical cancer in the post-HPV vaccination era. J. Public Health (Oxf.).

[bb0140] Drolet M., Boily M.C., Greenaway C., Deeks S.L., Blanchette C. (2013). Sociodemographic inequalities in sexual activity and cervical cancer screening: implications for the success of human papillomavirus vaccination. Cancer Epidemiol. Biomark. Prev..

[bb0050] Freeman HP, Wingrove BK (2005) Excess Cervical Cancer Mortality: A Marker for Low Access to Health Care in Poor Communities. Rockville, MD: National Cancer Institute, Center to Reduce Cancer Health Disparities. NIH Pub. No. 05–5282. Available at: http://crchd.cancer.gov/attachments/excess-cervcanmort.pdf Accessed April 1 2015.

[bb0015] Harper D.M., Nieminen P., Paavonen J., Lehtinen M. (2010). Cervical cancer incidence can increase despite HPV vaccination. Lancet Infect. Dis..

[bb0145] Healthy People 2020. C-15. Increase the proportion of women who receive a cervical cancer screening based on the most recent guidelines. Available at: http://healthypeople.gov/2020/topicsobjectives2020/DataDetails.aspx?hp2020id=C-15 Accessed April 1, 2015

[bb0170] Henderson L., Clements A., Damery S., Wilkinson C., Austoker J. (2011). ‘A false sense of security’? Understanding the role of the HPV vaccine on future cervical screening behaviour: a qualitative study of UK parents and girls of vaccination age. J. Med. Screen..

[bb0040] Huh W.K., Ault K.A., Chelmow D., Davey D.D., Goulart R.A., Garcia F.A., Kinney W.K., Massad L.S., Mayeaux E.J., Saslow D., Schiffman M., Wentzensen N., Lawson H.W., Einstein M.H. (Jan 6 2015). Use of primary high-risk human papillomavirus testing for cervical cancer screening: Interim clinical guidance. Gynecol. Oncol..

[bb0090] IBM Corp. (Released 2011). IBM SPSS Statistics for Windows, Version 20.0. Armonk, NY: IBM Corp.52. IBM Corp.(2012) Cochran-Armitage test for trend for 2xl tables. [Internet] Available at: http://www-01.ibm.com/support/docview.wss?uid = swg21480127 Accessed April 1, 2015.

[bb0130] (July 18, 2015). http://www.ncin.org.uk/publications/data_briefings/cervical_incidence_and_screening.

[bb0020] Kulasingam S.L., Pagliusi S., Myers E. (2007). Potential effects of decreased cervical cancer screening participation after HPV vaccination: an example from the US. Vaccine.

[bb0045] Lewin M.E., Lewin M.E., Altman S. (2000). Managed Care and the Future Viability of Safety Net Providers Committee on the Changing Market, Institute of Medicine. America's Health Care Safety Net: Intact but Endangered.

[bb0175] Leyden W.A., Manos M.M., Geiger A.M., Weinmann S., Mouchawar J. (2005). Cervical cancer in women with comprehensive health care access: attributable factors in the screening process. J. Natl. Cancer Inst..

[bb0035] Moyer V.A., on behalf of the U.S. Preventive Services Task Force. (2012) Screening for Cervical Cancer: U.S. Preventive Services Task Force Recommendation Statement. Ann Intern Med. 2012;156:880-891. Internet reference available at: *Screening for Cervical Cancer*. U.S. Preventive Services Task Force. http://www.uspreventiveservicestaskforce.org/uspstf/uspscerv.htm Accessed April 1, 2015. 10.7326/0003-4819-156-12-201206190-00424 22711081

[bb9000] Pollock K.G., Kavanagh K., Potts A., Love J., Cuschieri K. (2014). Reduction of low- and high-grade cervical abnormalities associated with high uptake of the HPV bivalent vaccine in Scotland. Br. J. Cancer.

[bb0160] Reddy D.M., Wasserman S.A. (1997). Patient anxiety during gynecologic examinations. Behavioral indicators.. J. Reprod. Med..

[bb0110] Sauer A.G., Jemal A., Simard E.P., Fedewa S.A. (Jun 5 2015). Differential uptake of recent Papanicolaou testing by HPV vaccination status among young women in the United States, 2008–2013. Cancer Epidemiol..

[bb0060] Scarinci I.C., Garcia F.A., Kobetz E., Partridge E.E., Brandt H.M. (2010). Cervical cancer prevention: new tools and old barriers. Cancer.

[bb0165] Snodgrass R., Naugler C. (2014). Use of the Papanicolaou test in women under 25 years of age in Southern Alberta. J. Obstet. Gynaecol. Can..

[bb0080] Solomon D., Davey D., Kurman R., Moriarty A., O'Connor D. (2002). The 2001 Bethesda System: terminology for reporting results of cervical cytology. Workshop.

[bb0095] StatSoft, Inc. (2013). STATISTICA (data analysis software system), version 12. Available at: www.statsoft.com. Accessed April 1, 2015.

