# Supplementary material for: Adherence to cervical cancer screening varies by human papillomavirus vaccination status in a high-risk population
Source: Prev Med Rep. 2015 Jul 31;2:711–6. doi: 10.1016/j.pmedr.2015.07.011 (PMC4721469; doi:10.1016/j.pmedr.2015.07.011)
Supplement: Supplementary Table — Vaccinated vs. non-vaccinated age and health care campus matched female descriptors. [file mmc1.doc]

Supplementary Table. Vaccinated vs. non-vaccinated age and health care campus matched females descriptors

|  | Non-vaccinated | Vaccinated |  |
| --- | --- | --- | --- |
|  | N=1154 | N=1154 | p-value |
| Race/Ethnicity, n (%) |  |  |  |
| White | 369 (32) | 446 (39) | <0.001 |
| Black | 642 (56) | 587 (51) | <0.01 |
| Hispanic | 75 (6) | 77 (7) | NS |
| Other | 68 (6) | 44 (4) | <0.05 |
| Gravidity, n (%) |  |  |  |
| n=0 | 163 (20) | 272 (25) | <0.01 |
| n≥1 | 657 (80) | 820 (75) |  |
| Parity, n (%) |  |  |  |
| n=0 | 217 (27) | 312 (29) | NS |
| n≥1 | 600 (73) | 780 (71) |  |
